# Alterations of the Transcriptome of *Sulfolobus acidocaldarius* by Exoribonuclease aCPSF2

**DOI:** 10.1371/journal.pone.0076569

**Published:** 2013-10-07

**Authors:** Birgit Märtens, Fabian Amman, Salim Manoharadas, Lukas Zeichen, Alvaro Orell, Sonja-Verena Albers, Ivo Hofacker, Udo Bläsi

**Affiliations:** 1 Max F. Perutz Laboratories, Department of Microbiology, Immunobiology and Genetics, Center of Molecular Biology, University of Vienna, Vienna, Austria; 2 Institute for Theoretical Chemistry, University Vienna, Vienna, Austria; 3 Molecular Biology of Archaea, Max-Planck Institute for Terrestrial Microbiology, Marburg, Germany; 4 Department of Computer Science and Interdisciplinary Center for Bioinformatics, University of Leipzig, Leipzig, Germany; University of Illinois, Urbana-Champaign, United States of America

## Abstract

Recent studies identified a 5´ to 3´ exoribonuclease termed Sso-RNase J in the crenarchaeon *Sulfolobus solfataricus* (Sso), which has been reclassified to the aCPSF2 (archaeal cleavage and polyadenylation specificity factor 2) group of β-CASP proteins. In this study, the Sso-aCPSF2 orthologue of *Sulfolobus acidocaldarius* (Saci-aCPSF2) was functionally characterized. Like Sso-aCPSF2, Saci-aCPSF2 degrades RNA with 5´ to 3´ directionality *in vitro*. To address the biological significance of Saci-aCPSF2, a deletion mutant was constructed, and the influence of Saci-aCPSF2 on the transcriptome profile was assessed employing high throughput RNA sequencing. This analysis revealed 560 genes with differential transcript abundance, suggesting a considerable role of this enzyme in RNA metabolism. In addition, bioinformatic analyses revealed several transcripts that are preferentially degraded at the 5´ end. This was exemplarily verified for two transcripts by Northern-blot analyses, showing for the first time that aCPSF2 proteins play a role in 5' to 3' directional mRNA decay in the crenarchaeal clade of Archaea.

## Introduction

RNA stability control is employed by cells to regulate gene expression and to adjust the level of protein synthesis in response to physiological needs. In all domains of life mRNA decay can commence in 5´ to 3´ as well as in 3´ to 5´ direction. In Eukaryotes and Bacteria, the stability of mRNA is affected by modifications of the 5´-end. In Eukaryotes, the removal of the 5´-end 7-methylguanosine cap and of the poly (A) tail at the 3´-end is considered rate-limiting for mRNA decay [1]. The removal of these modifications facilitates the degradation of mRNA by exoribonucleases [2]. In Bacteria, a triphosphorylated 5´-end and / or a 5´-terminal stem-loop structure counteract mRNA degradation by RNases. In *E. coli*, the decay of mRNA transcripts is initiated by the removal of the 5´-pyrophosphate by the enzyme RppH [3,4]. Following removal of the 5´-pyrophosphate, the 5’-end-dependent endoribonuclease RNase E can bind to the 5´-monophosphorylated ends of mRNAs, and can cleave the mRNA downstream [5]. The intermediate cleavage products are further degraded by 3´ to 5´-exonucleases including RNase R, PNPase, RNase II and oligoribonuclease [6]. In contrast, no orthologue of RNase E has been found in *Bacillus subtilis. B. subtilis* possesses RNase J1 and its paralogue RNase J2 that have endonucleolytic cleavage specificity similar to RNase E on some substrates [7]. In addition, they also encompass 5´ to 3´ exoribonuclease activity with a preference for monophosphorylated or hydroxylated 5´-ends [8]. RNase J1 belongs to the family of β-CASP metallo-β-lactamases, comprising three domains: a β-lactamase, a β-CASP and an N- or C-terminal extension with the catalytic site located between the β-lactamase core and the β-CASP domain [9]. *B. subtilis* RNase J combines endo- and exoribonucleotytic activities in a single protein and both functions are carried out by the same catalytic site [10].

Different RNases with endo- and exonucleolytic activity have been described in Archaea, but only a few of them have been shown to be involved in mRNA degradation [7,11-13]. We have recently identified in *Sulfolobus solfataricus* (Sso) an exoribonuclease with 5´ to 3´ directionality belonging to the β-CASP protein family of metallo-β-lactamases termed Sso-RNase J [14]. The enzyme, comprising four β-lactamase and three β-CASP motifs, has been recently reclassified to the aCPSF2 group of β-CASP proteins [9]. The members of this group are commonly restricted to the β-CASP and metallo-β-lactamase core domains, contain approximately 420 amino acids and no additional N- or C-terminal extensions [9]. Sso-aCPSF2 represents an exception within this group as it contains a ~ 40 amino acid N-terminal region. Sso-aCPSF2 is affected by the phosphorylation state of the 5´ end of RNA, and in contrast to other archaeal β-CASP proteins [15,16] requires Mg^+2^ ions for activity [14]. Although Sso-aCPSF2 was identified as the first archaeal 5´ to 3´ exonuclease, its role in crenarchaeal RNA metabolism remained elusive.

In this study we characterized the Sso-aCPSF2 orthologue of *S. acidocaldarius* (Saci). We show that the recombinant enzyme degrades RNA with 5´ to 3´ directionality *in vitro*. As Saci is more amenable to genetic manipulation than Sso, a deletion mutant of the corresponding Saci *2362* reading frame was constructed to unravel an *in vivo* role for aCPSF2 exoribonucleases in crenarchaeal RNA metabolism. A comparative analysis of the transcript abundance in Saci MW001 and in Saci MW001∆*2362* revealed that the Saci-aCPSF2 enzyme plays a major role in Saci mRNA turnover. Moreover, bioinformatic analyses together with biochemical studies identified transcripts that are degraded with 5´ to 3´ directionality, indicating for the first time *in vivo* that this pathway is operative in the crenarchaeal clade of Archaea.

## Results

### Saci-aCPSF2 exhibits 5´ to 3´ directional exoribonuclease activity *in vitro*

The Sso-aCPSF2 orthologue of Saci has been recently identified [14]. In contrast to the Sso-and *S. islandicus* (Sisl) aCPSF2 proteins, the Saci-aCPSF2 lacks the N-terminal extension and is composed of only the β-CASP and metallo-β-lactamase core-domains (Figure S1), thus representing a prototype member of this group. The Saci-aCPSF2 shares 55.5% and 55.9% amino acid identity with Sso-aCPSF2 (70.9% similarity) and Sisl-aCPSF2 (71.4% similarity), respectively, and the residues implicated in formation of the active site are highly conserved (Figure S1).

**Figure 1:**
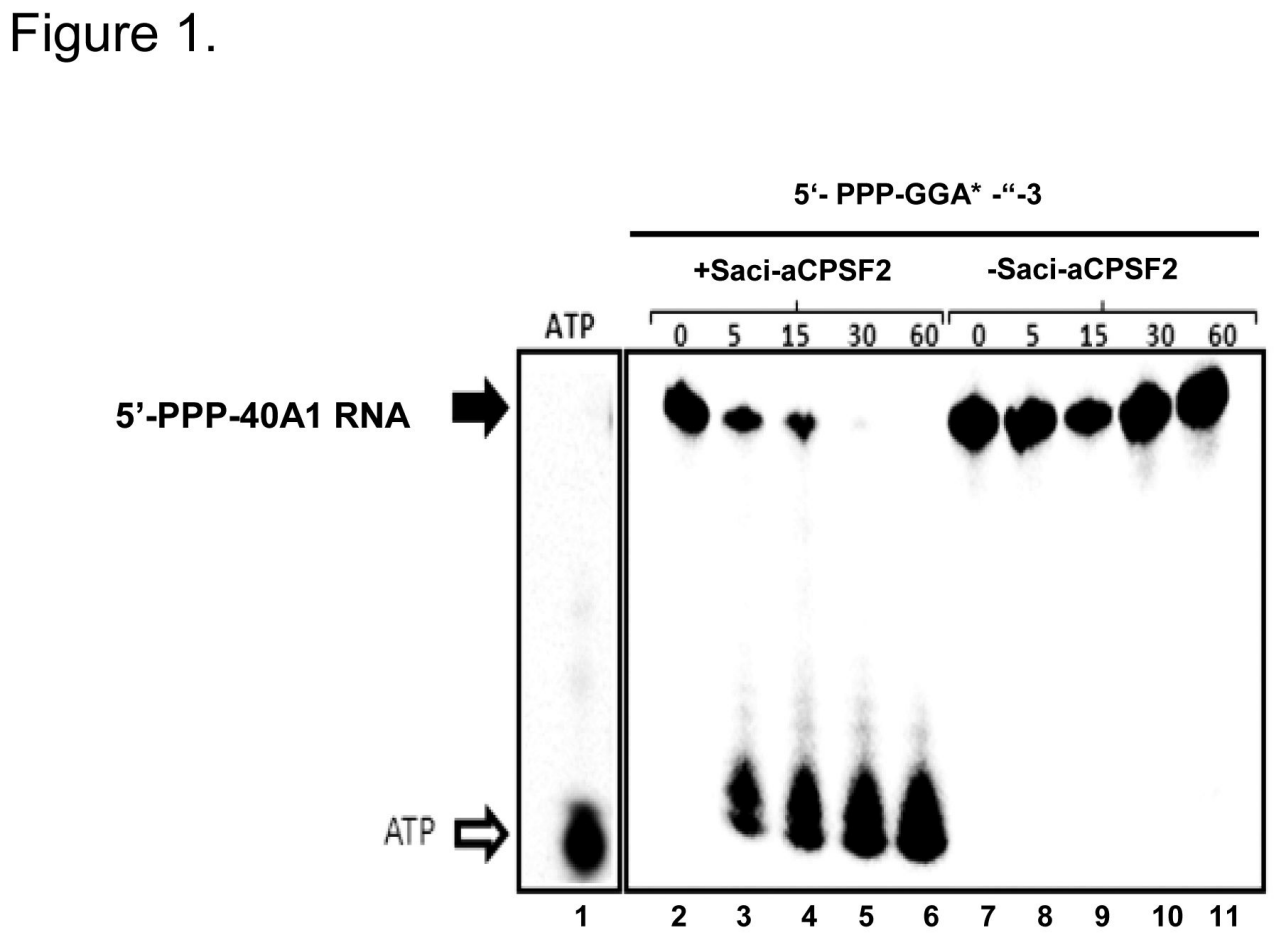
Saci-aCPSF2 displays 5´ to 3´ exonuclease activity. Lane 1, [α-^32^P]ATP was loaded on the gel. 5´-PPP-40A1 labeled RNA was incubated for 0´ to 60´ at 65°C in the presence of 500 ng recombinant Saci-aCPSF2 (lanes 2-6) or in the absence of the enzyme (lanes 7-11). The PPP-40A1 substrate contains a single labeled adenosine residue at position +3 (top; Table S2).

To test the enzymatic activity of Saci-aCPSF2, an *in vitro* degradation assay was performed with recombinant Saci-aCPSF2 purified from *E. coli* by means of the His-tag technology. A 42-nt-long synthetic RNA (termed 5´-PPP-40A1) harboring a 5`-tri-phosphate and a single radioactively labeled A nucleotide at the 5´-end was used as substrate. As anticipated, degradation of the 5´-PPP-40A1 RNA was observed in the presence of Saci-aCPSF2 (Figure 1, lanes 2-6), whereas the RNA remained stable over time in the absence of the enzyme (Figure 1, lanes 7-11). As shown in Figure 1 (lanes 2-6), the Saci-aCPSF2- mediated decay of 5´-PPP-40A1 RNA resulted in either a single nucleotide or short oligoribonucleotides, indicating an exoribonucleolytic activity. Moreover, as observed before for Sso-aCPSF2, the activity of Saci-aCPSF2 was dependent on the presence of Mg^+2^ ions (not shown), again indicating that Saci-aCPSF2 has similar enzymatic properties as the Sso orthologue [14].

To verify the apparent 5´ to 3´ exoribonuclease activity of Saci-aCPSF2, a 5´ end protection assay was performed. Translational initiation factor aIF2 of Sso was previously shown to bind *via* its γ-subunit to the 5´-triphosphate terminus of RNA, and thereby impede 5´ to 3´ directional RNA decay *in vitro* and *in vivo* [14,17]. Accordingly, we anticipated that binding of aIF2(γ) to the 5’-end of the RNA would reduce 5´ to 3´ directional decay by Saci-aCPSF2. To test this, 5´-PPP-40A1 RNA was used and the decay of the RNA by Saci-aCPSF2 was monitored in the presence and absence of aIF2(γ). As shown in Figure S2, when a/IF2(γ) was bound to the 5’-end of the RNA, the RNA was protected from 5´ to 3´ directional decay by Saci-aCPSF2, confirming the 5´-end dependent activity of the enzyme.

### Saci-aCPSF2 is involved in mRNA turnover

**Figure 2:**
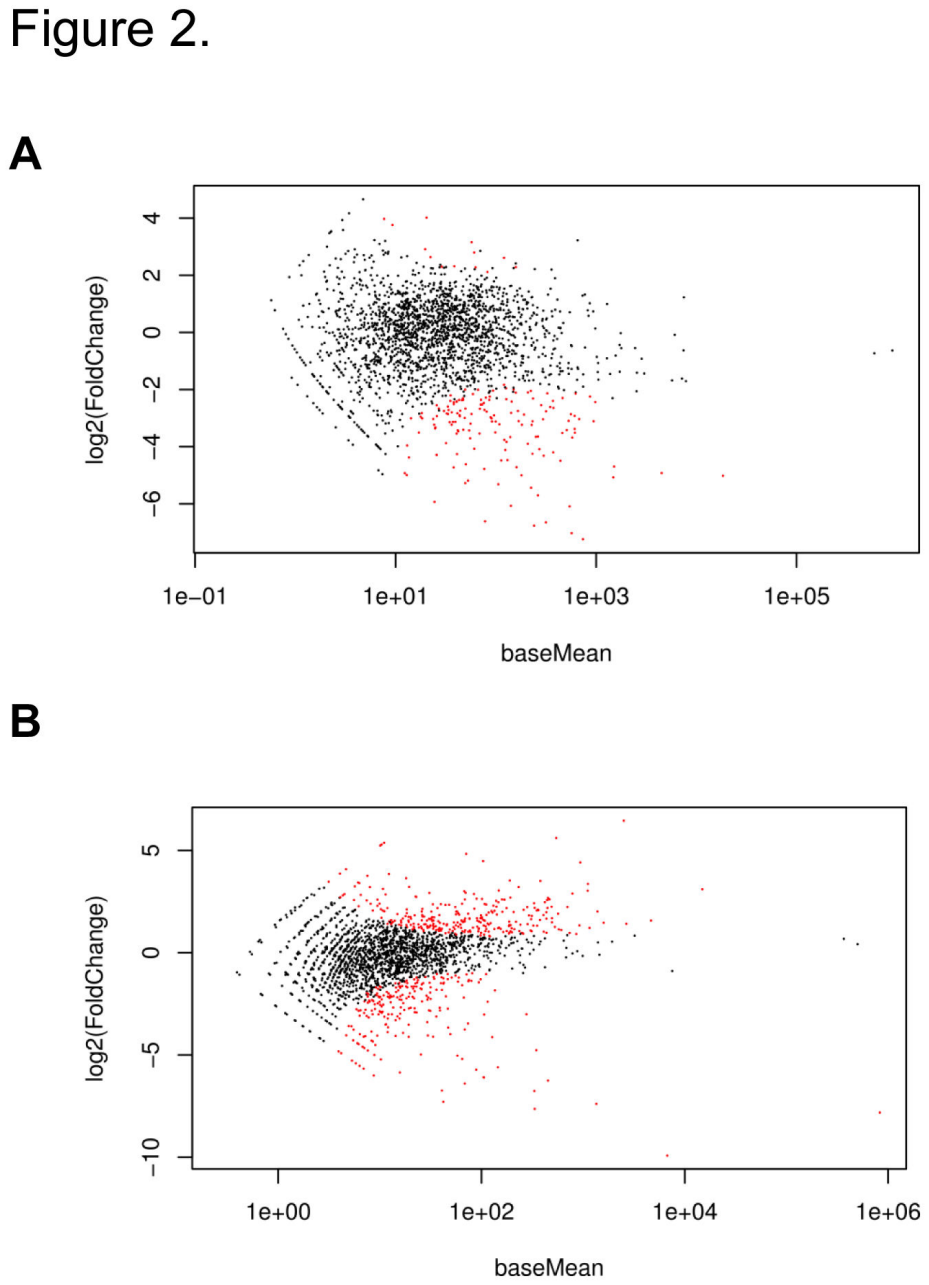
DESeq analysis of MW001 versus MW001*∆2362*. (**A**) Differential abundance of transcripts in MW001 and in MW001*∆2362* during logarithmic growth (each dot represents one transcript). (**B**) Differential abundance of transcripts in MW001 and MW001∆*2362* in stationary phase. The log_2_ fold-change is plotted against the mean expression level for each transcript. Red dots represent transcripts whose abundance is significantly changed (p-value adjusted for multiple testing < 0.1).

To obtain evidence for a role of Saci-aCPSF2 in mRNA turnover, the transcriptomes of the Saci strains MW001 and MW001*∆2362* (*∆*aCPSF2) were compared during logarithmic growth and in stationary phase using high throughput RNA sequencing (RNA-seq). Interestingly, the abundance of the Saci *2362* transcript, encoding Saci-aCPSF2, was higher during exponential growth than in stationary phase (Figure S3). In the mutant strain MW001*∆2362*, Saci *2362* mRNA was not detected, again confirming the deletion of the gene (not shown). All genes, annotated in the NCBI database were included in the differential gene expression analysis (Table S3; Table S4). Figure 2 depicts the log_2_-fold change versus the mean expression for all analyzed genes. When compared with strain MW001, 171 and 496 transcripts were differentially abundant in MW001*∆2362* during logarithmic growth and during stationary phase, respectively. Among them, the level of 107 mRNAs was significantly altered during both growth phases, logarithmic and stationary phase (not shown). In total, 560 unique transcripts showed a significant change in abundance during logarithmic (Table S3) and in stationary phase (Table S4), respectively. It seems worth noting that during logarithmic growth, when the Saci *2362* transcript abundance was increased, most of the differentially abundant transcripts are present at reduced levels in strain MW001. At variance, during stationary phase, when the Saci *2362* transcript levels were lower, the abundance of a considerable number of transcripts was found to be increased in strain MW001 when compared to MW001*2362*. Taken together, the transcriptome profiles of both strains implicated Saci-aCPSF2 in mRNA turnover.

### Saci-aCPSF2 is involved in 5´ to 3´ directional decay *in vivo*

**Figure 3:**
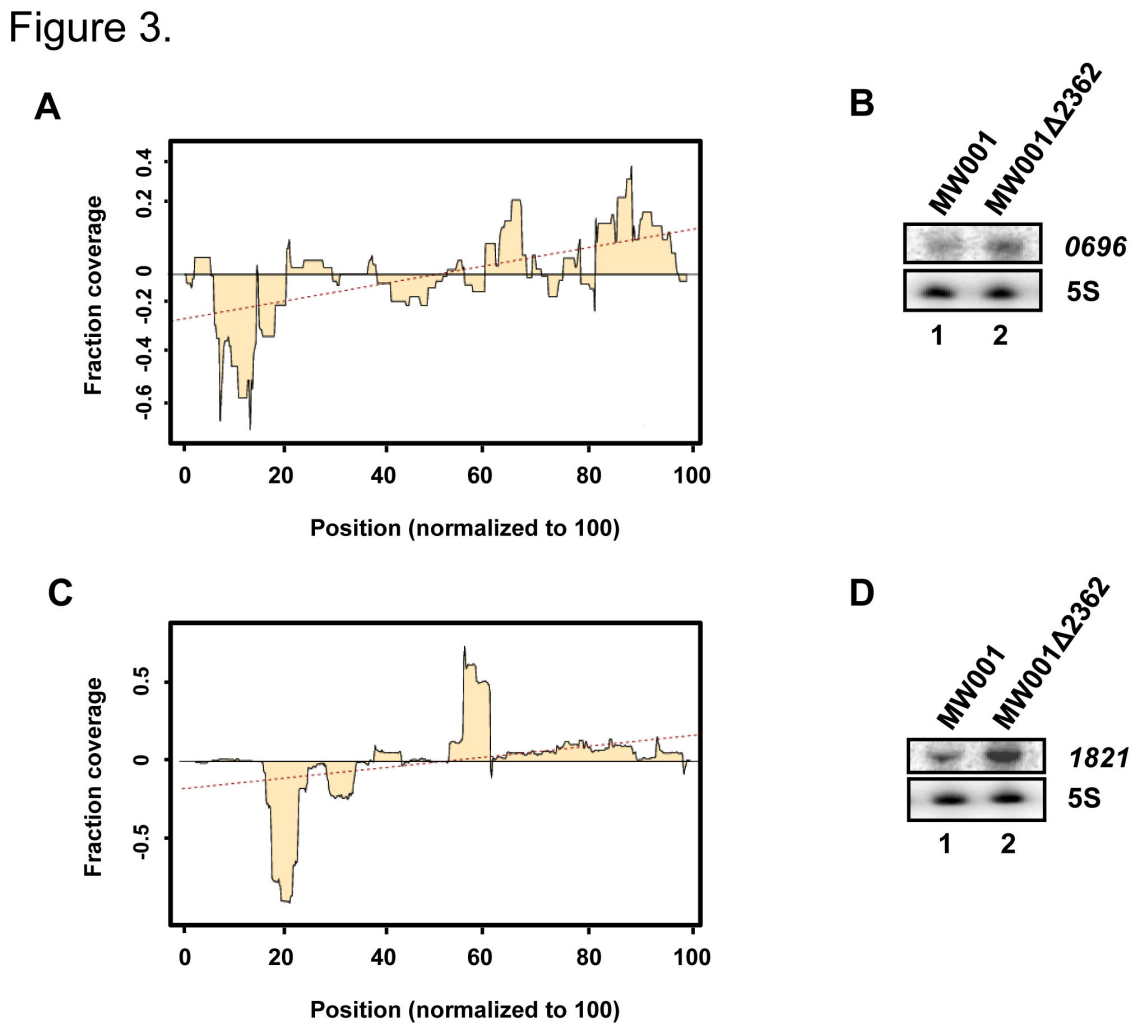
Preferential 5´-end degradation of transcripts. Read distribution over transcripts Saci *0696* (**A**) and Saci *1821* (**B**) *Normalized* “MW001 coverage” minus “MW001*∆2362* coverage” was plotted and a regression line was calculated. The slope of the regression line represents the coverage change of the 5´-end relative to the 3´-end in the mutant and in the wild-type. Preferential degradation from the 5´-end of the transcript corresponds to a positive slope of the regression line. The slope was calculated with 0.0037 and 0.0086 for *0696* and *1821*, respectively. (**C**, **D**) Detection of the steady state levels of the *0696* (**C**) and *1821* (**D**) transcripts by Northern-blot analysis. The transcript levels were determined either during logarithmic growth (**C**) or in stationary phase (**D**) of strain MW001 and strain MW001Δ*2362*, respectively. The result of one representative experiment is shown. The signals obtained from three independent experiments were quantified with ImageQuant software and averaged. The levels of the *0696* and *1821* transcripts were ~ 2- and ~ 3-fold increased in strain MW001Δ*2362* when compared with strain MW001, respectively.

As Saci-aCPSF2 is a 5´ to 3´ exoribonuclease, we next made attempts to identify transcripts preferentially degraded at the 5´-end. For each position along a transcript the read coverage was determined and normalized with the total read count of the whole transcript. This relative read coverage was further normalized to a transcript length of 100. Then, for each position the relative read coverage in the mutant was subtracted from the read coverage obtained for the wild-type strain. The resulting curve was approximated by a linear regression line, whose slope describes a relative read coverage shift between the mutant and the wild-type within one transcript. When compared with a given transcript isolated from MW001*∆2362* a decreased number of “5´-end-reads” in the corresponding transcript from MW001 gives rise to a positive slope of the regression line. In other words, it was assessed whether the 5´-region of a given transcripts is more abundant in the mutant, and therefore probably predominantly degraded in 5´ to 3´ direction. In the next step, only transcripts were selected, which were more abundant in the mutant strain (log_2_ fold change ≥ mean plus the standard deviation), and which showed in addition a positive slope of the regression line (≥ mean plus the standard deviation). From these analyses, 14 and 13 transcripts emerged as potential targets of Saci-aCPSF2 during logarithmic growth and in stationary phase (Table 1). The low numbers of identified potential RNase substrates results from the combination of the above described independent and rather strict analysis, with a small, hence reliable overlap. In Figure 3, the bioinformatic analysis is exemplarily shown for two selected transcripts Saci *0696* and Saci *1821*, which displayed a differential abundance during logarithmic growth and in stationary phase.

**Table 1:** Saci transcripts displaying accelerated 5´ end decay in strain MW001 when compared with strain MW001∆*2362* during logarithmic growth and in stationary phase.

Gene (log. phase)	predicated function	Gene (stat. phase)	predicted function
Saci *0084*	50S ribosomal protein L18e	Saci *0158*	Short chain dehydrogenase
Saci *0401*	Cobalamin biosynthesis protein CbiG	Saci *0211*	Carbon nitrogen hydrolase like
Saci *0582*	30S ribosomal protein S8	Saci *0384*	Hypothetical protein
Saci *0595*	50S ribosomal protein L23	Saci *0673*	Hypothetical protein
Saci *0696*	Nucleoside diphosphate kinase	Saci *0730*	Like Pre-rRNA processing protein TSR3
Saci *0708*	5-formaminioimidazole-4-carboxamide-1-(beta)-D-ribofuranosyl 5’-monophosphate synthetase	Saci *0892*	Zn-finger protein of UPF0148 family
Saci *1119*	Carbon monoxide dehydrogenase subunit like	Saci *1212*	Like AbrB family of transcriptional regulator
Saci *1386*	Hypothetical protein	Saci *1515*	Hypothetical protein
Saci *1410*	Phosphotransferase like	Saci *1821*	DNA protection protein DPS
Saci *1524*	Monogalactosyldiacylglycerolsynthase like	Saci *1832*	Fe-S oxidoreductase like
Saci *1551*	ATPase complex like	Saci *2086*	Quinol oxidase like
Saci *1705*	dTDP-4-dehydrorhamnose reductase	Saci *2197*	Hypothetical protein
Saci *2153*	Acetyltransferase	Saci *2309*	Hypothetical protein
Saci *2158*	Hypothetical protein		

Saci *0696* encodes a probable nucleoside diphosphate kinase [18]. The *0696* transcript was ~ 2.3-fold more abundant during logarithmic growth in strain MW001*∆2362* when compared with strain MW001 (not shown). In addition, the slope of the regression line for the *0696* transcript was 0.0037 (Figure 3A), which is indicative for 5´ to 3´ exoribonucleolytic decay.

Saci *1821* encodes a DNA protection protein [18]. The *1821* transcript was ~ 10-fold more abundant in strain MW001*∆2362* when compared with strain MW001 in stationary phase of growth (not shown). In addition, the slope of the calculated regression line for the *1821* transcript was 0.0086 (Figure 3C), again indicative for 5´ to 3´ exoribonucleolytic decay.

To verify the RNA-seq data, we used probes complementary to the 5´ end of the *0696* and *1821* transcripts and determined their abundance by Northern-blot analysis during logarithmic growth and in stationary phase, respectively. As shown in Figure 3B, the level of the Saci *0696* transcript was ~ 2-fold higher in strain MW001∆*2362* when compared with strain MW001 during logarithmic growth. Similarly, the abundance of the Saci *1821* transcript was ~ 3-fold higher in strain MW001∆*2362* when compared with strain MW001 in stationary phase (Figure 3D). Taken the RNA-seq data together with the Northern-blot analyses these studies indicated that Saci-aCPSF2 is involved in 5´ to 3´ directional mRNA decay *in vivo*.

## Discussion

In this study, we have characterized the Saci-aCPSF2 5´ to 3´ exoribonuclease. Like Sso-aCPSF2 [14], Saci-aCPSF2 requires Mg^+2^ ions for activity. This could be a peculiar feature of the aCPSF2 group, as other archaeal β-CASP proteins are not Mg^+2^-dependent [9].

In *B. subtilis* RNase J a 5´ terminal triphosphorylated nucleotide cannot be accommodated in the mononucleotide binding pocket without placing the following phosphodiester bond out of phase with the catalytic center [10]. Taverniti et al. [19] have devised a model, wherein 5´ proximal endonucleolytic cleavage, and thus removal of the 5´ terminal triphosphorylated nucleotide precedes the subsequent 5´ to 3´ directional exonucleolytic decay of RNA by RNase J enzymes [19]. In addition to the mononucleotide, short oligoribonuleotides appeared as well after Saci-aCPSF2–mediated degradation (Figure 1). Thus, it is possible that in addition to its 5´ to 3´ exonuclease activity Saci-aCPSF2 can cleave also endonucleolytically close to the 5´ end as observed for RNase J of *M. smegmatis* and RNAse J1 of *B. subtilis* [19], which in turn might explain why Saci-aCPSF2 can degrade a RNA substrate with a 5´ triphosphate end as used in this study.

To assign a biological function to aCPSF2 enzymes in *Sulfolobales*, we have compared the transcriptome of Saci strain MW001 with that of strain MW001∆*2362*. Surprisingly, a large number of the identified transcripts showed an increased level in strain MW001 when compared with the mutant strain. In *B. subtilis*, the absence / decrease of RNases J1/J2 results in similar numbers of transcripts whose abundance is either increased or decreased, suggesting a complex role of these ribonucleases in both degradative and regulatory events [20]. It is conceivable that the altered levels of many transcripts in the presence or absence of a given RNase result from a perturbation of the expression levels of a limited number of regulatory genes, and thus from indirect effects. In any case, almost 50% of the detected transcripts displayed an increased level in MW001*∆2362*, indicating that Saci-aCPSF2 is involved in RNA degradation. Using the COG database tool [21] the function of the 560 affected mRNAs was classified. As shown in Figure S4, Saci-aCPSF 2 altered the levels of genes involved in many different functions. As mentioned above, many of the affected RNAs may not be direct substrates of Saci-aCPSF2, and it is therefore difficult to predict whether the enzyme affects particular pathways or functions in the cell.

In addition, the analyses of the data set revealed transcripts, which (i) are more abundant in strain MW001*∆2362* and (ii) for which a decreased read coverage of the 5´-end was observed during logarithmic growth and in stationary phase, respectively. The reduced abundance of the 5´ end was verified for two genes, Saci *0696* and Saci *1821*, by Northern-blot analysis, supporting the notion that Saci-aCPSF2 acts as a 5´ to 3´ exoribonuclease *in vivo*.

In *B. subtilis* the absence or decrease of both, RNase J1 and J2, alters the expression level of hundreds of genes [20]. In contrast, the effect on global gene expression was moderate in single mutant strains, suggesting that two nucleases have largely overlapping substrates specificities [20]. The growth rate of MW001 and MW001*∆2362* was indistinguishable in Brock’s medium (not shown). As Saci-aCPSF2 is obviously not essential, we asked whether other β-CASP proteins exist in *S. acidocaldarius*. A homology search using blastp (http://blast.ncbi.nlm.nih.gov) for *S. acidocaldarius* proteins identified Saci *0639* (Figure S5). In contrast to Saci-aCPSF2, this protein displays typical signatures of the aCPSF 1 group of β-CASP proteins [9]. It contains an N-terminal KH domain, a metallo-β-lactamase domain followed by the β-CASP domain, and motif 2 is conserved (Figure S5). Clearly this finding poses the question as to the function of the other β-CASP protein in *S. acidocaldarius*. Similar to the RNases J1/2 in *B. subtilis*, this enzyme could form a complex with Saci-aCPSF2 and could jointly affect mRNA turnover [22].

## Materials and Methods

### Strains and plasmids

All *E. coli* strains were routinely grown in Luria-Bertani broth (LB), supplemented with appropriate antibiotics to maintain plasmids. The archaeal strains were grown in Brock’s medium [23] supplemented with 0.2% arabinose and 0.1% NZamine or 0.1% tryptone at 75°C. In addition, 10 µg/ml uracil was added. The pH was adjusted to 2-3 with sulfuric acid. The bacterial / archaeal strains, plasmids and media used in this study are listed in Table S1.

### Purification of Saci-aCPSF2 and Sso-aIF2(γ)

The Saci ORF *2362*, encoding Saci-aCPSF2, was PCR amplified with primers A72_FP and B72_RP (Table S2) using genomic DNA as template. The PCR product was cleaved with NcoI and XhoI and cloned into plasmid pET28b (Table S1), resulting in plasmid pET28b-Saci*2362*. Recombinant Saci-aCPSF2 was purified under denaturing conditions (8M urea) by Ni-NTA affinity chromatography (Qiagen) following standard protocols. To remove urea, the protein samples were dialyzed against different buffers (100 mM KCl, 50 mM Tris pH 7.0 containing 4M, 2M, 1M or no urea, respectively). The purified protein was stored at -80°C in the presence of 5% glycerol. The Sso aIF2γ-subunit was purified as previously described [24].

### *In vitro* synthesis of the RNA substrate

To characterize the enzymatic properties of Saci-aCPSF2, the 42-nt-long, synthetic RNA (5´-PPP-40A1 RNA with a tri-phosphate group at the 5´-end (PPP) was used in the assays. The RNA harbored a single radioactive labeled A nucleotide at position +3 at the 5´ end. The 5´-labeled RNA was synthesized as follows: The T7 oligonucleotide (Table S2) containing a T7-promoter was hybridized to the 40A1-oligonucleotide (Table S2). The duplex was used as template for *in vitro* transcription using the Ambion MEGAshortscript T7 Kit together with [α-^32^P]ATP. The RNA was then loaded on a 12% polyacrylamide gel containing 8M urea and purified following standard protocols.

### RNA degradation assays

The RNA degradation assays were carried out as recently described [14]. Saci-aCPSF2 activity was assayed in a 10 µl reaction volume containing 10 mM MgCl_2_, 10 mM KCl, 5 mM Tris pH 7.5, 0.25 µM of the RNA substrate and 500 ng of purified Saci-aCPSF2. The reaction mix was incubated for 0 to 60 min at 65°C. The reaction was terminated by addition of RNA-loading dye, containing 0,025% SDS and 0,05 mM EDTA followed by incubation on ice. The samples were then resolved on a 20% PAA / 7 M urea gel. The gel was subjected to autoradiography using a Typhoon 8600 PhosphorImager.

### Construction of the *S. acidocaldarius* MW001∆*2362* mutant

For construction of the Saci*∆2362* in frame deletion mutant, the up- (868 bp) and downstream (2145 bp) flanking regions of Saci ORF *2362* were PCR amplified from genomic DNA. To amplify the upstream region, the oligonucleotides Saci_2362_KO_Fw_up and Saci_2362_KO_Rv_up (Table S2) were used. For amplification of the downstream region the primers Saci_2362_KO_Fw_dwn and Saci_2362_KO_Rv_dwn (Table S2) were used. By overlap extension PCR [25], the up- and downstream flanking regions were joined by using the outward bound primer of the respective primer pair. These fragments were combined in a subsequent reaction in which the overlapping ends anneal, allowing the 3' overlap of each strand to serve as a primer for the 3' extension of the complementary strand. The resulting fusion product was amplified further by PCR. The PCR products were cleaved with PstI and BamHI, and subsequently ligated into plasmid pΔ2*pyr*EF, which contains the *pyr*EF cassette from Sso [26]. The ligation mixture was transformed in *E. coli* strain ER1821 (NEB) which results in hypermethylation of plasmid DNA. The resulting methylated `Saci∆*2362* deletion plasmid` was transformed into Saci MW001 by electroporation as described [27]. Briefly, 50 µl of electro-competent Saci MW001 cells were mixed with 100 ng or 300 ng of plasmid. The mixture was transferred to 0.1 cm electroporation cuvettes (Bio-Rad). The electroporation program of the Genepulser MXcell (Bio-Rad) was set as follows: 1500 Volt, 600 Ώ and 25 µF. After electroporation the cells were mixed with 50 µl of 2 x Brock’s medium and incubated at 75°C for 30 minutes with shaking. After incubation, the cells were seeded first on selection gelrite plates without uracil and incubated at 75°C for 5 to 6 days. Integrants were subsequently selected on 5-FOA (100 µg/ml) gelrite plates to allow the excision of the DNA region containing the target gene. The deletion mutant strain Saci MW001*∆2362* was confirmed by sequencing of the PCR product obtained with the primers KO_test FP and KO_test RP (Table S2).

### RNA sequencing

The strains MW001 and MW001∆*2362* were grown in Brock’s medium supplemented with 0.1% tryptone and 10 µg/ml uracil. For each transcriptome analysis, total RNA from logarithmically growing cultures (OD_600_ ~ 0,35) and cultures grown to stationary phase (OD_600_ ~ 1,0) were prepared as follows: Total RNA was isolated, using Trizol [28]. The samples were then treated with DNase I (DNase I, RNase-free, Roche Applied Science) and a control PCR was performed to confirm complete degradation of chromosomal DNA. The used primers (test_PCR_RP1/2, FP_1/2_) are listed in Table S2. The analyses included 2 biological replicates for each sample condition. The RNA was fragmented to an average length of 200-300 nt by incubation for 2 minutes at 94°C in 40 mM Tris-acetate pH 8.2, 100 mM potassium-acetate and 30 mM magnesium-acetate [29]. The samples were cooled on ice and purified on a Sephadex G50 column. The cDNA synthesis was carried out using the SuperScript® Double-Stranded cDNA Synthesis Kit (Invitrogen) following the manufacturer’s instructions. The cDNA was purified using phenol/CHCl_3_. The cDNA of the different samples were further processed and subjected to next generation RNA sequencing (NGS; Illumina platform GAIIx) at the CSF (http://www.csf.ac.at). The sequencing data can be found at the short read archive (http://www.ncbi.nlm.nih.gov/sra; accession numbers SRR942640 and SRR942641).

### Bioinformatic analyses

The obtained sequencing reads were mapped onto the genome of *Sulfolobus acidocaldarius* DSM 639 (NC_007181) using Segemehl (version 0.9.3) with default parameters [30]. For each annotated mRNA from the NCBI database the number of mapped reads, for each growth phase and separately for each of the two replicates, were determined by counting all reads with an overlap of at least 1 nt. To identify transcripts with differentially abundance in MW001 and MW001Δ*2362* grown either in logarithmic phase or grown to stationary phase, a differential gene expression analysis was performed using the tool DESeq (version 1.5) part of the bioconductor packages [31]. The determination of the abundance change of the 5´ end of transcripts relative to the 3´ end was performed as described above by self-implemented Perl and R scripts.

### Northern-blot analyses

Total RNA was prepared from strains MW001 and MW001*∆2362* during logarithmic growth (OD_600_ ~ 0.35) and when the cells reached stationary phase (OD_600_ ~ 1.0) using the Trizol method [28]. 10 µg of total RNA was transferred to a Nitrocellulose membrane (Amersham Hybond, GE healthcare) and UV-crosslinked. The membrane was pre-incubated with Roti-Hybri-Quick (Roth). The 5´ end labeled oligonucleotides (H83, N83 or Z83; Table S2) were added followed by incubation at 45°C for 12h. The membrane was washed with washing-solution I (2x SSC, 0,1% SDS) and washing-solution II (0,5x SSC, 0,1% SDS), and then subjected to autoradiography using a Typhoon 8600 PhosphorImager.

## Supporting Information

Figure S1 **Alignment of the aCPSF2 enzymes of *S. solfataricus* (Sso)*, Sulfolobus islandicus* (Sisl) and *S. acidocaldarius* (Saci) using CLC sequence viewer 6.6.2 software.** The conserved amino acid residues are depicted in red. (A) The β-CASP domain is highlighted with a black box. Residues boxed in green build the four β-lactamase motifs 1-4, whereas the three β-CASP motifs A, B and C are highlighted with a yellow box. (B) Motif 2 present in the catalytic domain of β-CASP proteins [9] is conserved in Sso-aCPSF2, Sisl-aCPSF2 and Saci-aCPSF2.
(TIF)

Figure S2 **The translation initiation factor aIF2(γ) impedes 5´ to 3´ degradation by Saci-CPSF2.** 5´-PPP-40A1 RNA (5 pmol) was incubated for 0 to 60 minutes at 65°C in the presence of Saci-aCPSF2 (500 ng), in the absence (lane 1-5) and in the presence (lanes 6-10) of Sso-a/eIF2 (γ) (25 pmol) bound to the 5´ triphosphate.
(TIF)

Figure S3 **Abundance of Saci 2362 mRNA in strain MW001 in logarithmic- (blue bars) and in stationary phase (red bars).** The values are an average of two biological replicates. The rpm* in the y-axis represents the reads per megareads (number of reads mapped to Saci *2362* divided by the total number of million reads in the sample).
(TIF)

Figure S4 **Functional classification of transcripts affected by Saci-CPSF2.** Each affected transcript was assigned to a certain class, using the COG database tool [21]..
(TIF)

Figure S5 **Alignment of Saci-aCPSF2 (Saci 2362) and Saci-aCPSF1 (Saci 0639) using CLC sequence viewer 6.6.2 software.** The conserved amino acid residues are depicted in red. The β-CASP domain is highlighted with a black box. Residues boxed in green build the four β-lactamase motifs 1-4, whereas the three β-CASP motifs A, B and C are highlighted with a yellow box. The domain structure of Saci-aCPSF2 and Saci-aCPSF1 is shown at the right.
(TIF)

Table S1 **Strains and Plasmids used in this study.**
(DOC)

Table S2 **Oligonucleotides used in this study.**
(DOC)

Table S3 **Differential abundance of transcripts in MW001 and MW001*∆2362* during logarithmic growth.**
(XLS)

Table S4 **Differential abundance of transcripts in MW001 and MW001*∆2362* in stationary phase.**
(XLS)
