# Supplementary figures and images for: Alterations of the Transcriptome of Sulfolobus acidocaldarius by Exoribonuclease aCPSF2
Source: PLoS One. 2013 Oct 7;8(10):e76569. doi: 10.1371/journal.pone.0076569 (PMC3792030; doi:10.1371/journal.pone.0076569)

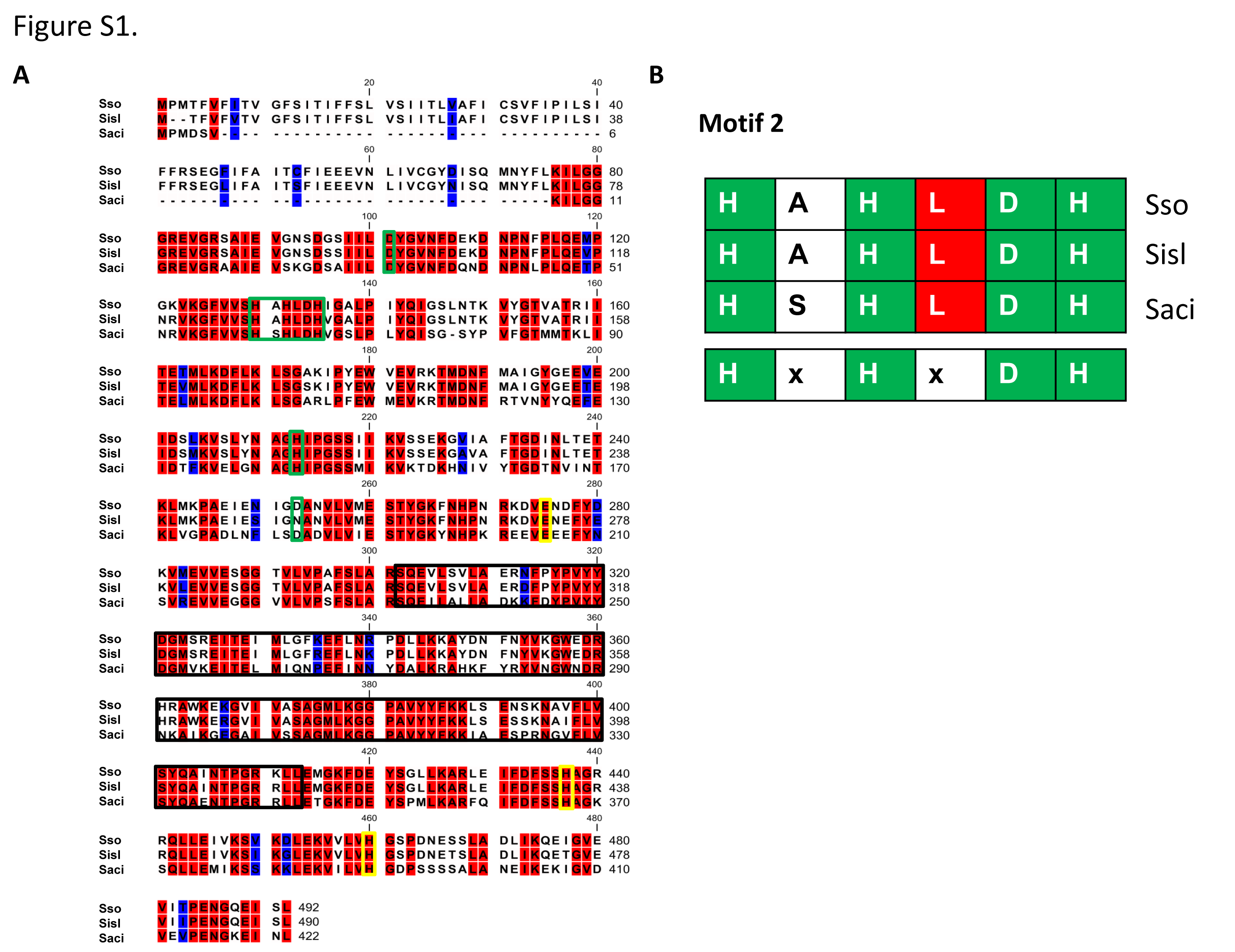

Supplement: Figure S1 — Alignment of the aCPSF2 enzymes of S. solfataricus (Sso), Sulfolobus islandicus (Sisl) and S. acidocaldarius (Saci) using CLC sequence viewer 6.6.2 software. The conserved amino acid residues are depicted in red. (A) The β-CASP domain is highlighted with a black box. Residues boxed in green build the four β-lactamase motifs 1-4, whereas the three β-CASP motifs A, B and C are highlighted with a yellow box. (B) Motif 2 present in the catalytic domain of β-CASP proteins [9] is conserved in Sso-aCPSF2, Sisl-aCPSF2 and Saci-aCPSF2. (TIF) [file pone.0076569.s001.tif]

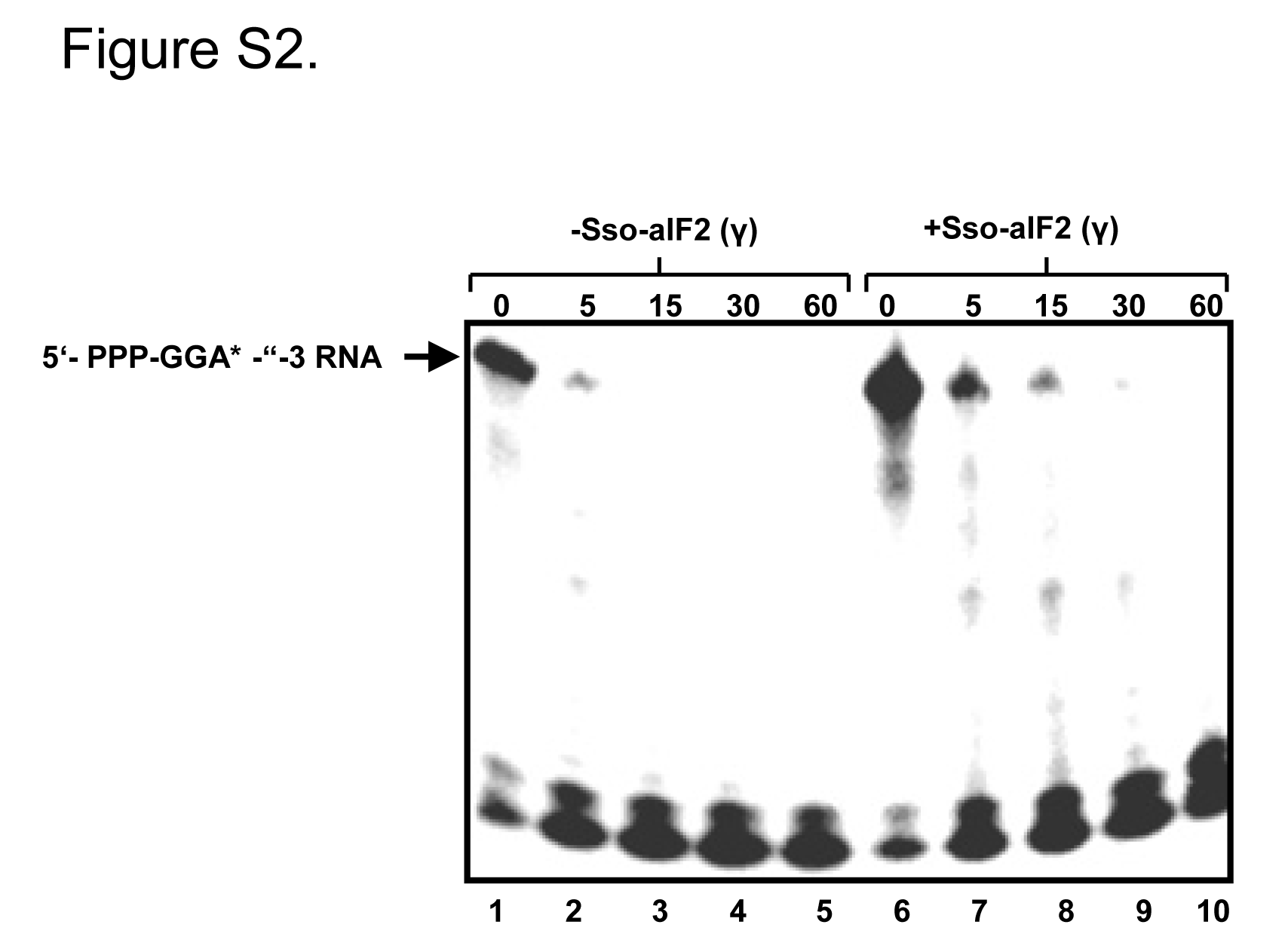

Supplement: Figure S2 — The translation initiation factor aIF2(γ) impedes 5´ to 3´ degradation by Saci-CPSF2. 5´-PPP-40A1 RNA (5 pmol) was incubated for 0 to 60 minutes at 65°C in the presence of Saci-aCPSF2 (500 ng), in the absence (lane 1-5) and in the presence (lanes 6-10) of Sso-a/eIF2 (γ) (25 pmol) bound to the 5´ triphosphate. (TIF) [file pone.0076569.s002.tif]

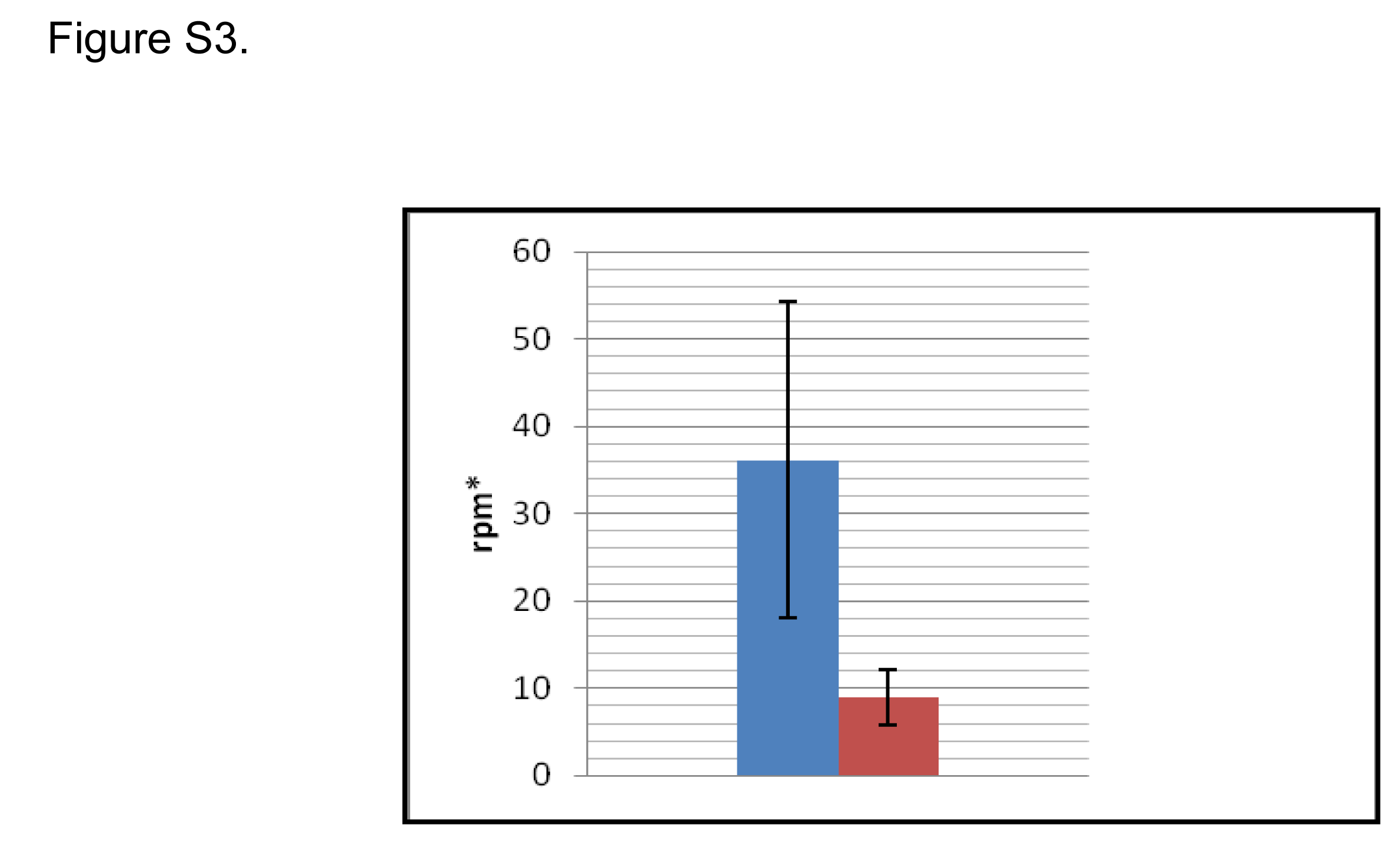

Supplement: Figure S3 — Abundance of Saci 2362 mRNA in strain MW001 in logarithmic- (blue bars) and in stationary phase (red bars). The values are an average of two biological replicates. The rpm* in the y-axis represents the reads per megareads (number of reads mapped to Saci 2362 divided by the total number of million reads in the sample). (TIF) [file pone.0076569.s003.tif]

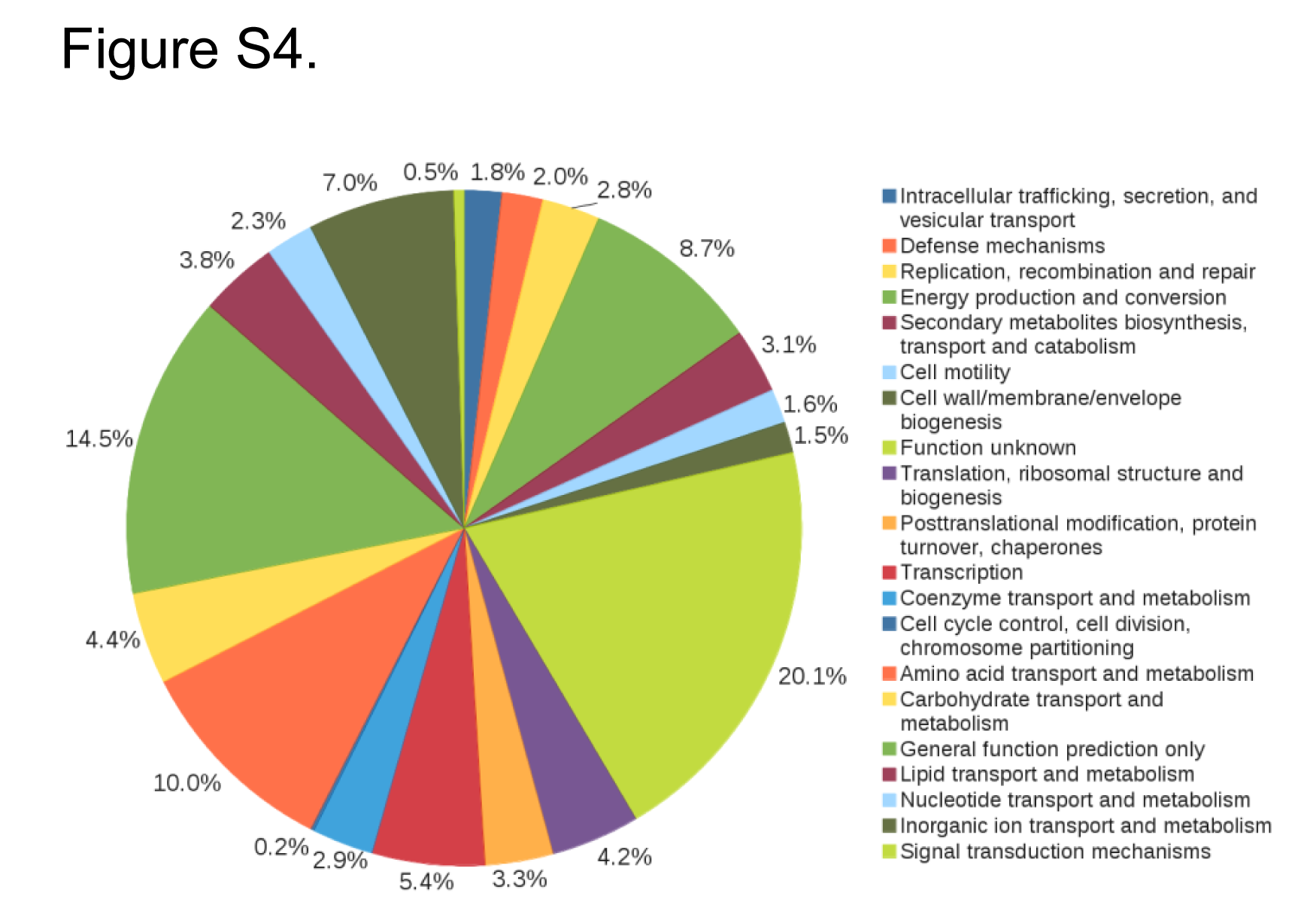

Supplement: Figure S4 — Functional classification of transcripts affected by Saci-CPSF2. Each affected transcript was assigned to a certain class, using the COG database tool [21].. (TIF) [file pone.0076569.s004.tif]

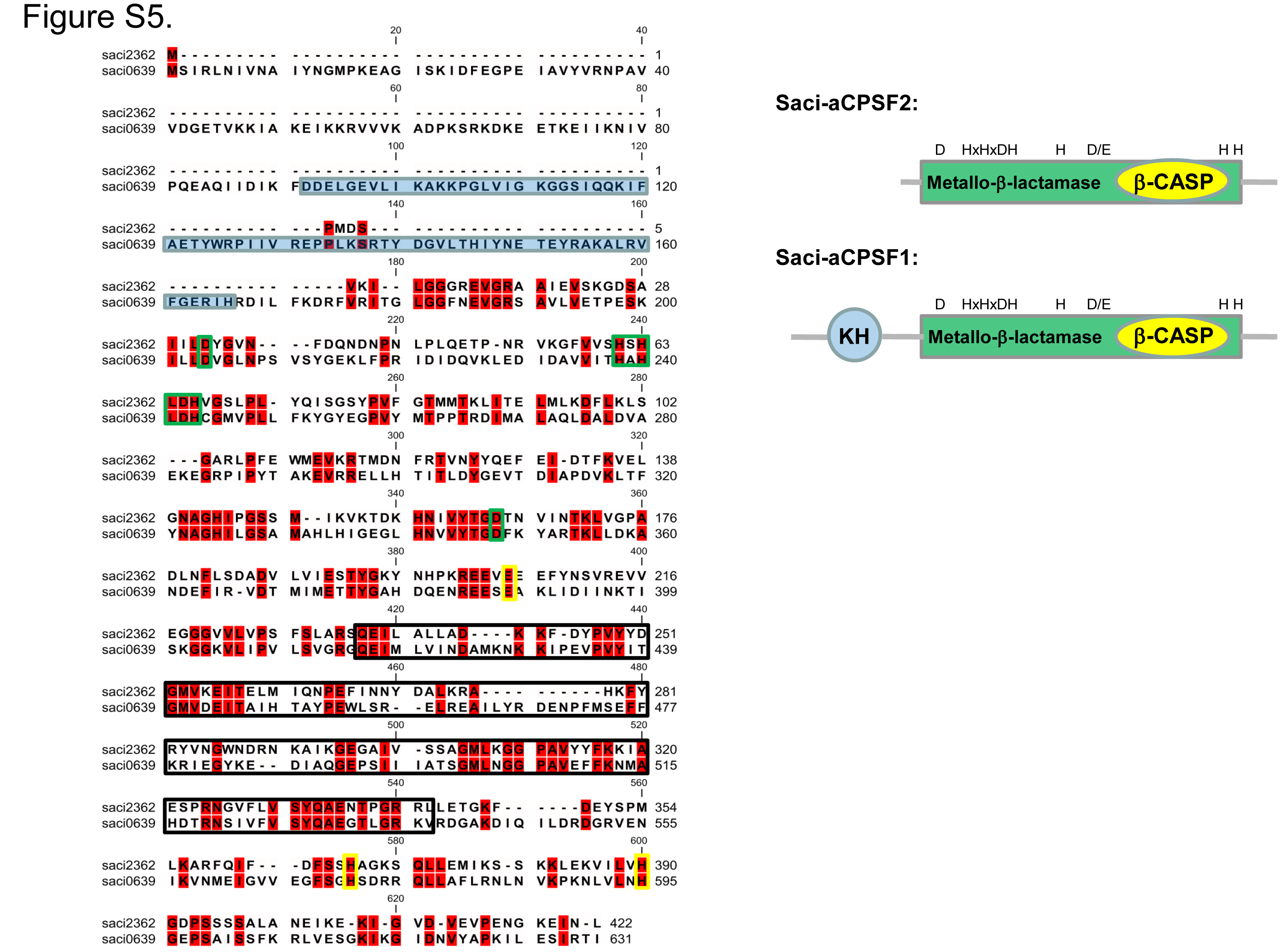

Supplement: Figure S5 — Alignment of Saci-aCPSF2 (Saci 2362) and Saci-aCPSF1 (Saci 0639) using CLC sequence viewer 6.6.2 software. The conserved amino acid residues are depicted in red. The β-CASP domain is highlighted with a black box. Residues boxed in green build the four β-lactamase motifs 1-4, whereas the three β-CASP motifs A, B and C are highlighted with a yellow box. The domain structure of Saci-aCPSF2 and Saci-aCPSF1 is shown at the right. (TIF) [file pone.0076569.s005.tif]
