# Supplementary material for: Alterations of the Transcriptome of Sulfolobus acidocaldarius by Exoribonuclease aCPSF2
Source: PLoS One. 2013 Oct 7;8(10):e76569. doi: 10.1371/journal.pone.0076569 (PMC3792030; doi:10.1371/journal.pone.0076569)
Supplement: Table S1 — Strains and Plasmids used in this study. (DOC) [file pone.0076569.s006.doc]

**Table S1 Strains and plasmids used in this study**

| ***Sulfolobus* strains** | **Genotype** | **Source** |
| --- | --- | --- |
| *Sulfolobus acidocaldarius*  MW001 | *pyrEF^-^*, *lacS* | S. Albers, strain collection |
| *Sulfolobus acidocaldarius* MW001*∆2362* | *pyrEF^+^*,*lacS*, *∆2362* | This study |
| ***E. coli* strains** | **Genotype** | **Source** |
| BL21 *(Rosetta)* | F^-^*, dcm, ompT, hsdS(rB-mB-), galλ*(DE3) | Stratagene |
| *E. coli ER1821* | F^-^ *glnV44 e14*^-^(McrA^-^) *rfbD1? relA1? endA1 spoT1? thi-1* Δ(mcrC-mrr)*114::IS10* | New England Bio Labs |
| **Plasmids** | **Description** | **Source** |
| pET28b | T7 promoter, His-tag coding sequence, MCS, *lacI* coding sequence, (Kan^R^) | Novagen |
| pET28b-Saci*2362* | encoding Saci-aCPSF2 with C-terminal His-tag | This study |
| p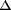2*pyr*EF | pBluescript-based vector with *S. solfataricus pyr*EF gene | S. Albers, plasmid collection |
| Saci *2362* deletion plasmid | pΔ2*pyr*EF-vector containing Saci *2362* deletion cassette | S. Albers, plasmid collection |
