# Supplementary material for: Alterations of the Transcriptome of Sulfolobus acidocaldarius by Exoribonuclease aCPSF2
Source: PLoS One. 2013 Oct 7;8(10):e76569. doi: 10.1371/journal.pone.0076569 (PMC3792030; doi:10.1371/journal.pone.0076569)
Supplement: Table S2 — Oligonucleotides used in this study. (DOC) [file pone.0076569.s007.doc]

**Table S2.** Oligonucleotides used in the study.

| **Name** | **Sequence** | | | | **Destination** | | **Coordinates** | |  |
| --- | --- | --- | --- | --- | --- | --- | --- | --- | --- |
| **A72_FP** | 5’-TTCTTCCATGGCTATGGATTCAGTGAAGTACTGGGC-3’ | | | | *Saci_2362* forward primer, cleavage site for *Nco*I (underlined) | | 2209571-2209596 | |  |
| **B72_RP** | 5’-AAGAACTCGAGTAGGTTAATCTCCTTACCGTTTTCCG-3’ | | | | *Saci_2362* reverse primer, cleavage site for *Xho*I (underlined) | | 2210807-2210832 | |  |
| **40A1-oligonucleotide (5´-end)** | 5’-AGACAGAAACCACAGAACGAGACAGAAACCACAGAACGTCCTATAGTGAGTCGTATTAC-3’ | | | | For synthesis of RNA 40A1 labeled at 5’ end | | |  |  |
| **5´-PPP-40A1RNA sequence** | 5’-GG**A**CGUUCUGUGGUUUCUGUCUCGUUCUGUGGUUUCUGUCU-3’ | | | | Substrate for Saci-aCPSF2 (labeled at the 5´-end (A) | | |  |  |
| **T7-oligo** | 5’-GGGCTCTAGAGTAATACGACTCACTATAGG-3’ | | | | Containing a T7-promoter for *in vitro* transcription | | |  |  |
| **Saci_2362_ KO_Fw_up_ *Pst*I** | 5´-GTACTGCAGCTTCGACCCTTCTCGTAATC-3´ | | | | upstream flanking region, forward primer, cleavage site for *Pst*I (underlined) | | | 2208701-2208720 |  |
| **Saci_2362_KO _Rv_up** | 5´-GAAGAATTATCATTAATTCAACCGCCAAAC-3´ | | | | upstream flanking region, reverse primer | | | 2209550-2209569 | |
| **Saci_2362_ KO_Fw_dwn** | 5´-CGGTTGAATTAATGATAATTCTTCTAATTC-3´ | | | | downstream flanking region, forward primer | | | 2209556-210850 | |
| **Saci_2362_KO_Rv_dwn_ *Bam*HI** | 5´-GATGGATCCTGCGTCCTGTAGGTAATCAC-3´ | | | | downstream flanking region, reverse primer, cleavage site for *Bam*HI (underlined) | | | 2211682-2211701 | |
| **KO_test RP** | | 5’-CTACGAAGGGTATCCATTCC-3´ | | | | Primer anneals downstream of *Saci_2362* | 2211788-2211807 | | |
| **KO_test FP** | | 5’-AGGAAGCATTAAGGTGTAAC-3´ | | | | Primer anneals upstream of *Saci_2362* | 2208586-2208605 | | |
| **test_PCR_Saci RP1** | | | 5´-CTGTAAATCAGTGCTGTTACAAT-3´ | | | Test for the presence of chromosomal DNA | 1516600-1516622 | | |
| **test_PCR_Saci FP1** | | | 5´-GGAGGCTCATTGGTCGAATCCA-3´ | | | Test for the presence of chromosomal DNA | 1517148-1517169 | | |
| **test_PCR_Saci RP2** | | | 5´-AGTCGGGATTCCAGTCTGAAATG-3´ | | | Test for the presence of chromosomal DNA | 1618994-1619016 | | |
| **test_PCR_Saci FP2** | | | | 5´-CCATCTGTTCAATTCTCTGCTA-3´ | | Test for the presence of chromosomal DNA | 1619801-1619823 | | |
| **H83** | | | | 5’-TTCCCCTACTAATCTTCTTCTTACACCGTCTG-3’ | | Detection of 5´end of Saci_0696 | 556656-556642 | | |
| **N83** | | | | 5‘-TCTAAACCAGATTTTTCCAATATTTCCACTCCAACAG-3‘ | | Detection of 5`end of Saci_1821 | 1589557-1589594 | | |
| **Z83** | | | | 5’-GAGCGGCTTAACTTCCGGGT-3’ | | Detection of 5S rRNA | 1294084-1294101 | | |
